# Effects of hyaluronan treatment on lipopolysaccharide-challenged fibroblast-like synovial cells

**DOI:** 10.1186/ar2104

**Published:** 2007-01-10

**Authors:** Kelly S Santangelo, Amanda L Johnson, Amy S Ruppert, Alicia L Bertone

**Affiliations:** 1 Department of Clinical Sciences, College of Veterinary Medicine, The Ohio State University, 1900 Coffey Road, Columbus OH 43210, USA; 2 Center for Biostatistics, The Ohio State University, 320 West 10th Avenue, Columbus OH 43210, USA

## Abstract

Numerous investigations have reported the efficacy of exogenous hyaluronan (HA) in modulating acute and chronic inflammation. The current study was performed to determine the *in vitro *effects of lower and higher molecular weight HA on lipopolysaccharide (LPS)-challenged fibroblast-like synovial cells. Normal synovial fibroblasts were cultured in triplicate to one of four groups: group 1, unchallenged; group 2, LPS-challenged (20 ng/ml); group 3, LPS-challenged following preteatment and sustained treatment with lower molecular weight HA; and group 4, LPS-challenged following pretreatment and sustained treatment with higher molecular weight HA. The response to LPS challenge and the influence of HA were compared among the four groups using cellular morphology scoring, cell number, cell viability, prostaglandin E_2 _(PGE_2_) production, IL-6 production, matrix metalloproteinase 3 (MMP3) production, and gene expression microarray analysis. As expected, our results demonstrated that LPS challenge induced a loss of characteristic fibroblast-like synovial cell culture morphology (*P *< 0.05), decreased the cell number (*P *< 0.05), increased PGE_2 _production 1,000-fold (*P *< 0.05), increased IL-6 production 15-fold (*P *< 0.05), increased MMP3 production threefold (*P *< 0.05), and generated a profile of gene expression changes typical of LPS (*P *< 0.005). Importantly, LPS exposure at this concentration did not alter the cell viability. Higher molecular weight HA decreased the morphologic change (*P *< 0.05) associated with LPS exposure. Both lower and higher molecular weight HA significantly altered a similar set of 21 probe sets (*P *< 0.005), which represented decreased expression of inflammatory genes (PGE_2_, IL-6) and catabolic genes (MMP3) and represented increased expression of anti-inflammatory and anabolic genes. The molecular weight of the HA product did not affect the cell number, the cell viability or the PGE_2_, IL-6, or MMP3 production. Taken together, the anti-inflammatory and anticatabolic gene expression profiles of fibroblast-like synovial cells treated with HA and subsequently challenged with LPS support the pharmacologic benefits of treatment with HA regardless of molecular weight. The higher molecular weight HA product provided a cellular protective effect not seen with the lower molecular weight HA product.

## Introduction

Hyaluronan (HA), a common component of connective tissue, is a long, unbranched nonsulfated glycosaminoglycan essential for the normal function of diarthrodial joints. The high concentration (2.5–4 mg/ml) of HA in synovial fluid is maintained by lining type B fibroblasts and is composed of a polydispersed population with molecular weights that vary from 2 × 10^6 ^to 1 × 10^7 ^Da [1]. These large molecules can form extensive macromolecule networks, although the nature of these associations and their orientation is not resolved [2,3]. It is postulated that hydrophobic regions of these complexes provide sites for interactions with cell membranes and other phospholipids [4]. The identification of specific receptors to which HA binds – specifically cluster determinant 44, intercellular adhesion molecule 1, and the receptor for hyaluronan-mediated motility – on a diverse number of cells supports the pharmacologic activity of HA in addition to its rheologic properties [5,6]. HA can also readily enter cells by endocytosis and can interact with intracellular proteins [7]. Receptor–HA binding results in the stimulation of signaling cascades that moderate cellular functions, particularly cell migration, proliferation, and endocytosis [8,9]. The unique properties of HA are equally important for providing nutrients to cartilage, eliminating metabolic byproducts and deleterious substances from the joint cavity, and maintaining overall joint homeostasis by inhibiting phagocytosis, chemotaxis, scar formation, and angiogenesis [10,11].

Proinflammatory cytokines, free radicals, and proteinases found in pathologic conditions such as rheumatoid arthritis and osteoarthritis can adversely affect the type B synovial cells and lead to the synthesis of HA with abnormal molecular weight [12]. Furthermore, HA may be directly depolymerized by free radicals, intracellular hyaluronidases, and other glycosidases found in diseased synovium [13]. The decrease in molecular size, in combination with dilution from inflammatory infiltration of plasma fluid and proteins in aberrant joint conditions, reduces the rheologic properties of synovial fluid [14]. Viscosupplementation, a procedure in which abnormal synovial fluid is removed and replaced with purified high molecular weight HA, was developed to combat these anomalous processes [15].

Numerous *in vitro *investigations have reported the efficacy of exogenous HA in modulating acute and chronic inflammation, either by reducing cellular interactions [16], binding mitogen-enhancing factors [17,18], or suppressing the production of proinflammatory mediators such as IL-1β [19,20]. *In vivo *studies have focused on the anti-inflammatory effects [21-23] and analgesic effects [24] of HA. Interestingly, positive clinical outcomes can be achieved with HA of both high and very low molecular weight [1], and studies have shown that the lubricating characteristics of HA in synovial joints are not dependent on the HA molecular weight [25]. The effects of HA on intracellular processes may depend on the molecular weight of the HA molecule that is interacting with receptors and promoting stable receptor clustering but a definitive mechanism has not been identified [26].

Lipopolysaccharide (LPS) induces characteristic and well-defined inflammatory processes and degradation cascades in synovial tissue *in vitro *[27-29] and *in vivo *[30,31], and induces gene expression alterations in other articular cells *in vitro *[32,33]. LPS also plays an important role as an adjuvant in the stimulation of autoimmune arthritis in rodents [34]. To gain a better understanding of the intracellular signaling events triggered by HA of differing molecular weights, this study used cellular morphology, prostaglandin E_2 _(PGE_2_) production, IL-6 production, matrix metalloproteinase 3 (MMP3) production, and a species-specific microarray to elucidate the global gene changes of fibroblast-like synovial cells treated with commercial intra-articular joint supplements prior to LPS challenge. Our goal was to identify the protective effects of HA against LPS and to determine whether these effects were dependent on the molecular weight of the HA administered. We hypothesized that higher molecular weight HA would improve the negative cellular changes associated with LPS challenge.

## Materials and methods

### Animals, tissue harvest and cell culture

The study protocol as described was granted ethics approval by The Ohio State University Institutional Animal Care and Use Committee. Three clinically normal adult horses 8–15 years of age were selected for the study based on normal physical and gait evaluations. Tarsocrural joints were deemed normal on the basis of palpation and gross observation at tissue harvest. Synovium was collected from the dorsomedial pouch of both tarsocrural joints and pooled within each animal after aseptic preparation and opening of the joint. Cells were isolated and grown in culture until >95% confluent and were subsequently stored in a standard cryopreservation solution (10% dimethylsulfoxide/90% fetal bovine serum) at -80°C as primary cultures. Fibroblast-like synovial cells for each animal were thawed, resuspended in Dulbecco's modified Eagle medium supplemented with 10% fetal bovine serum, 29.2 mg/ml L-glutamine, 50 U penicillin/ml, and 50 U streptomycin/ml (DMEM-S), and were grown in monolayer under standard sterile conditions until >95% confluent (~24 hours) in Cellstar T75 flasks (Greiner Bio-One, Longwood, FL, USA). It was anticipated that fibroblast-like synovial cells were the dominant cell population in the cultures; no inflammatory cells were present. All flasks appeared to have similar, if not identical, cell populations and densities at this point.

### Experimental design

Fibroblast-like synovial cells >95% confluent (day 0) were allocated in triplicate to one of four groups: group 1, unchallenged; group 2, LPS-challenged; group 3, LPS-challenged following pretreatment and sustained treatment with lower (5 × 10^5^–7.5 × 10^5 ^Da) molecular weight HA (Bioniche Animal Health, Pullman, WA, USA) at 10 mg/ml; and group 4, LPS-challenged following pretreatment and sustained treatment with higher (3 × 10^6 ^Da) molecular weight HA (Pfizer Animal Health, New York, NY, USA) at 5 mg/ml.

Pretreatment with HA on day 0 was as follows: group 3 received 3 ml (equivalent to one dose) of lower molecular weight HA diluted in 12 ml DMEM-S, and group 4 received 3 ml (equivalent to one manufacturer's recommended dose) of higher molecular weight HA diluted in 12 ml DMEM-S. Groups 1 and 2 received 15 ml DMEM-S only.

On day 1, groups 2, 3 and 4 received a 2-hour challenge with 3 ml LPS from *Escherichia coli *055:B5 (Sigma Chemical, St Louis, MO, USA) at a concentration of 20 ng/ml followed by three washes/dilutions with Gey's balanced salt solution and replacement of appropriate media by group assignment. On day 2 the media were collected and frozen at -80°C and the cells were isolated for RNA extraction.

### Cellular morphology and cell count

Flasks were evaluated microscopically in triplicate for each horse and for each group on days 0–2. Morphology scores for the fibroblast-like synovial cells were assigned at three random locations throughout the center of the culture flasks at four specific time points: day 0, before the initial product application; day 1, prior to LPS challenge; day 1, immediately following 2-hour LPS challenge; and day 2, immediately prior to cell collection. Morphology scores were assigned based on the following scale: 0, >95% attached with healthy fibroblastic morphology; 1, 5–25% rounded and detached; 2, 26–50% rounded and detached; 3, 51–75% rounded and detached; and 4, >76% rounded and detached.

### Enzyme-linked immunosorbent assays

The concentrations of PGE_2_, IL-6, and MMP3 in cell culture media from each horse at day 2 were determined using commercial competitive ELISAs (R&D Systems, Minneapolis, MN, USA). All assays were performed according to the manufacturer's protocols. The optical density of each sample was read by the Ultra Microplate Reader EL808 (Bio-Tek Instruments, Winooski, VT, USA) and expressed as picograms per milliliter. Prior to running the experimental samples, it was confirmed that neither HA product significantly interfered with the activity of the assays. Briefly, two sets of standards were created: the first was made using standards as described by the manufacturer's protocol, and the second was made using these same standards following the addition of the appropriate HA product (at concentrations described above). The resulting optical densities were compared using a paired *t*-test analysis and no significant difference was detected.

### Microarray analysis

Total RNA was isolated from the remaining cell pellet using the phenol–chloroform extraction technique (Invitrogen, Carlsbad, CA, USA) as described by the manufacturer's protocol. RNA of the highest quality from each animal and from each treatment group was used for species-specific microarray analysis. Sample concentration and purity were measured by use of UV spectra (260 nm and 280 nm) and were confirmed using the Agilent 2100 Bioanalyzer (Agilent Technologies, Palo Alto, CA, USA). All protocols were conducted in accordance with the manufacturer's instructions (Affymetrix, Santa Clara, CA, USA). For processing, total RNA (5 μg) was reverse transcribed into double-stranded cDNA using RT/polymerase and the T7-(dT)24 primer. Biotinylated cRNA was synthesized by *in vitro *transcription and the cRNA products were fragmented prior to hybridization overnight at 45°C for 16 hours on an equine-specific gene expression microarray representing 3,098 unique genes, all of which have been annotated [35]. Microarrays were washed at low-stringent and high-stringent conditions and were stained with streptavidin–phycoerythrin in accordance with established protocol. The microarray design (accession number A-AFFY-81) and the experiment submission (accession number E-MEXP-940) are available online in EBI's ArrayExpress public repository [36].

### Validation of microarray by real-time RT-PCR

Fibroblast-like synovial cells from three individual horses were grown under cell culture conditions as described above until they were >95% confluent. At this point, individual culture flasks from each horse were exposed to Gey's Balanced Salt Solution (unchallenged control), 100 ng/ml LPS, or 1,000 ng/ml LPS for 2 hours. Cells were immediately collected for RNA extraction as described above; total RNA from each horse was pooled within each of the three treatment groups. Microarray analysis proceeded as previously described. PCR primers for three genes expected to have great, modest, and minimal responses to LPS challenge based on previous work [35] (IL-1α, TNFα, and prostaglandin peroxide synthase, respectively) were designed for real-time RT-PCR using the Primer Express Software (Applied Biosystems, Foster City, CA, USA). Two-step RT-PCR using the SYBR^® ^Green PCR Master Mix was performed according to the manufacturer's protocol and universal thermal cycling parameters (Applied Biosystems). Care was taken to ensure that DNA contamination was not present in the samples. The fold changes of each gene for each LPS-challenged group were calculated relative to the unchallenged control.

### Statistical analyses

Objective and scored data were compared using two-way analysis of variance followed by pair-wise comparisons with a Bonferroni correction. Repeated-measures analysis was performed on the cell counts and the cell morphology data. Statistical significance was set at α = 0.05. Microarray data were analyzed using dChip version 1.3 [37] (Harvard University, Cambridge, MA, USA). Array normalization was performed using the invariant set procedure; model-based expression indices were computed using only perfect-match probes. Probe-set level data identified as array outliers by dChip were omitted and considered missing data in subsequent analyses. The model-based expression indices values were then exported to BRB ArrayTools version 3.2.3 for further analysis (National Cancer Institute, Bethesda, MD, USA).

Paired *t *tests compared gene expression between the unchallenged control group (group 1) and the LPS-challenged control group (group 2). A two-way analysis of variance compared gene expression among the LPS-challenged control group (group 2) and the two HA-treated groups (groups 3 and 4), blocking on the horse. For the probe sets showing differential expression among the three LPS-challenged groups (*P *< 0.005), pair-wise comparisons were performed and *P *values were adjusted by Holm's method. All tests involving gene expression data used a random variance model [38]. The statistical analyses for the microarray data as described above are included in the experiment submission available online in ArrayExpress [36].

## Results

### Cellular morphology, viability, and count

**Figure 1:**
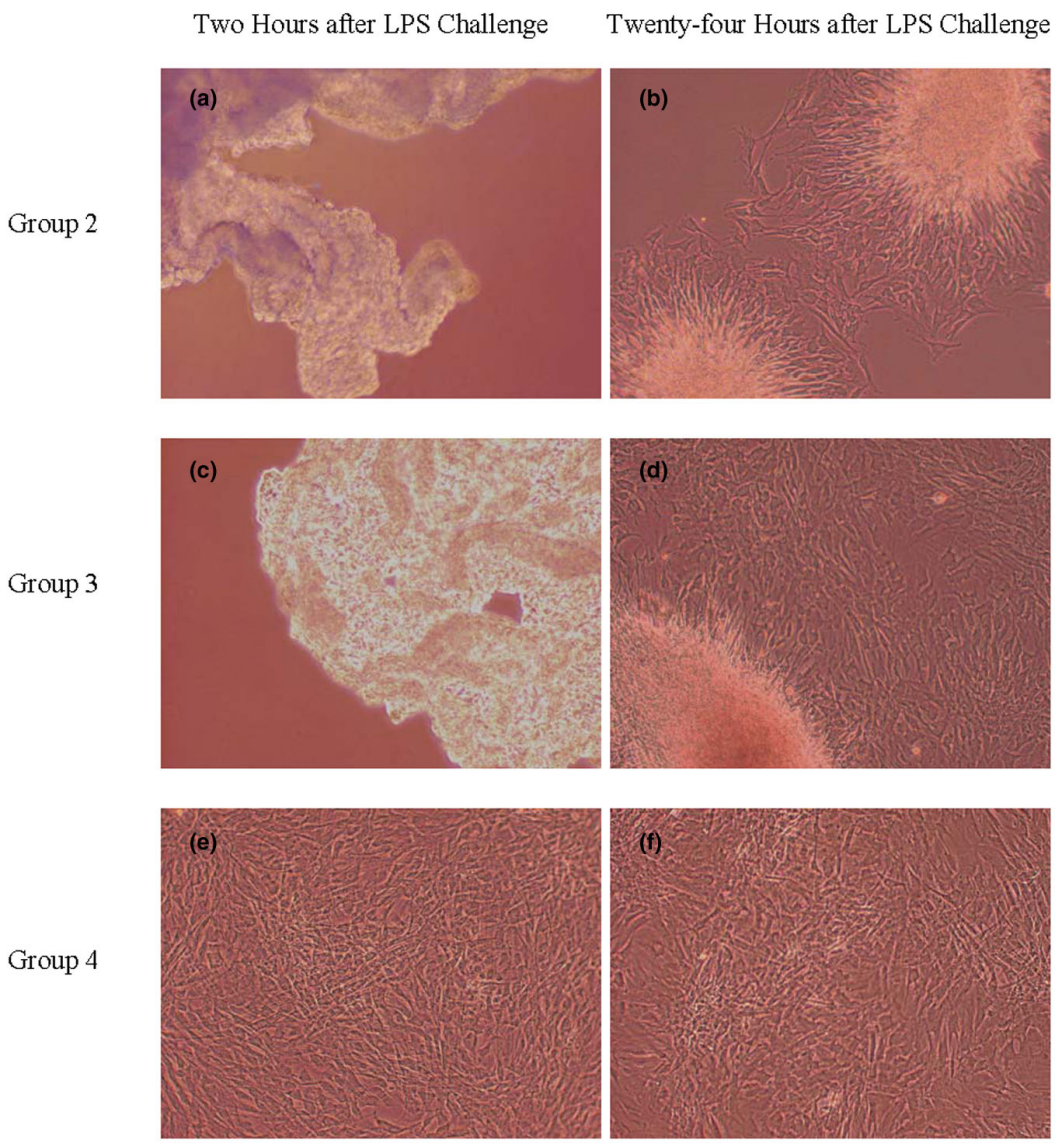
Representative microscopic images of fibroblast-like synovial cells post-lipopolysaccharide challenge. Representative microscopic images (400× magnification) of fibroblast-like synovial cells **(a)**, **(c)**, and **(e) **2 hours post-lipopolysaccharide (LPS) challenge and **(b)**, **(d)**, and **(f) **24 hours post-LPS challenge. Cells treated with the higher molecular weight hyaluronan (HA) product (group 4, pretreatment and sustained treatment with higher molecular weight HA) were protected from (d) and (e) the morphologic changes induced by LPS, including the loss of cell attachment to the culture flask and the pronounced cellular contraction that were seen in (a), (b) group 2 (LPS control) and (c), (d) group 3 (preteatment and sustained treatment with lower molecular weight HA).

The LPS-challenged control group (group 2) and the lower molecular weight HA-treated group (group 3) had significantly greater morphology scores than the unchallenged control group (group 1) or the higher molecular weight HA-treated group (group 4) (*P *< 0.05; Table 1). Morphologic changes in groups 2 and 3 included reversible loss of cell attachment to the culture flask and cell contraction (Figure 1).

Cell viability was high in all groups and no difference was found among the groups (Table 1), indicating that 20 ng/ml LPS did not kill a significant number of cells. Cell counts in the LPS-challenged groups 2, 3, and 4 were significantly lower than those of the unchallenged control group (group 1) (*P *< 0.05; Table 1).

### Prostaglandin E_2_, IL-6, and MMP3 ELISAs

Expression of inflammatory products, particularly PGE_2_, was anticipated to increase in response to LPS challenge [39-41]. As expected, there was a greater than 1,000-fold increase in the PGE_2 _concentration in the cell culture media of groups 2, 3, and 4 compared with that in group 1 (*P *< 0.05). There was also a significant difference between the concentrations of PGE_2 _produced by groups 3 and 4 relative to group 2 (*P *< 0.05; Table 2). There was not a significant difference in PGE_2 _production between groups 3 and 4.

Two genes, IL-6 and MMP3, were common to the two separate gene expression analyses described below (see Gene expression profiling). Commercial competitive ELISAs were performed to confirm the trends seen with the microarray data. For both genes, protein levels in group 2 were greater than those in groups 1, 3, and 4 (*P *< 0.01). There was no statistical difference in protein levels among groups 1, 3, and 4 (Table 2).

### Microarray validation by real-time RT-PCR

Consistent and comparable fold changes in gene expression were found between the microarray data and real-time RT-PCR for the three genes of interest (Table 3).

### Gene expression profiling

**Figure 2:**
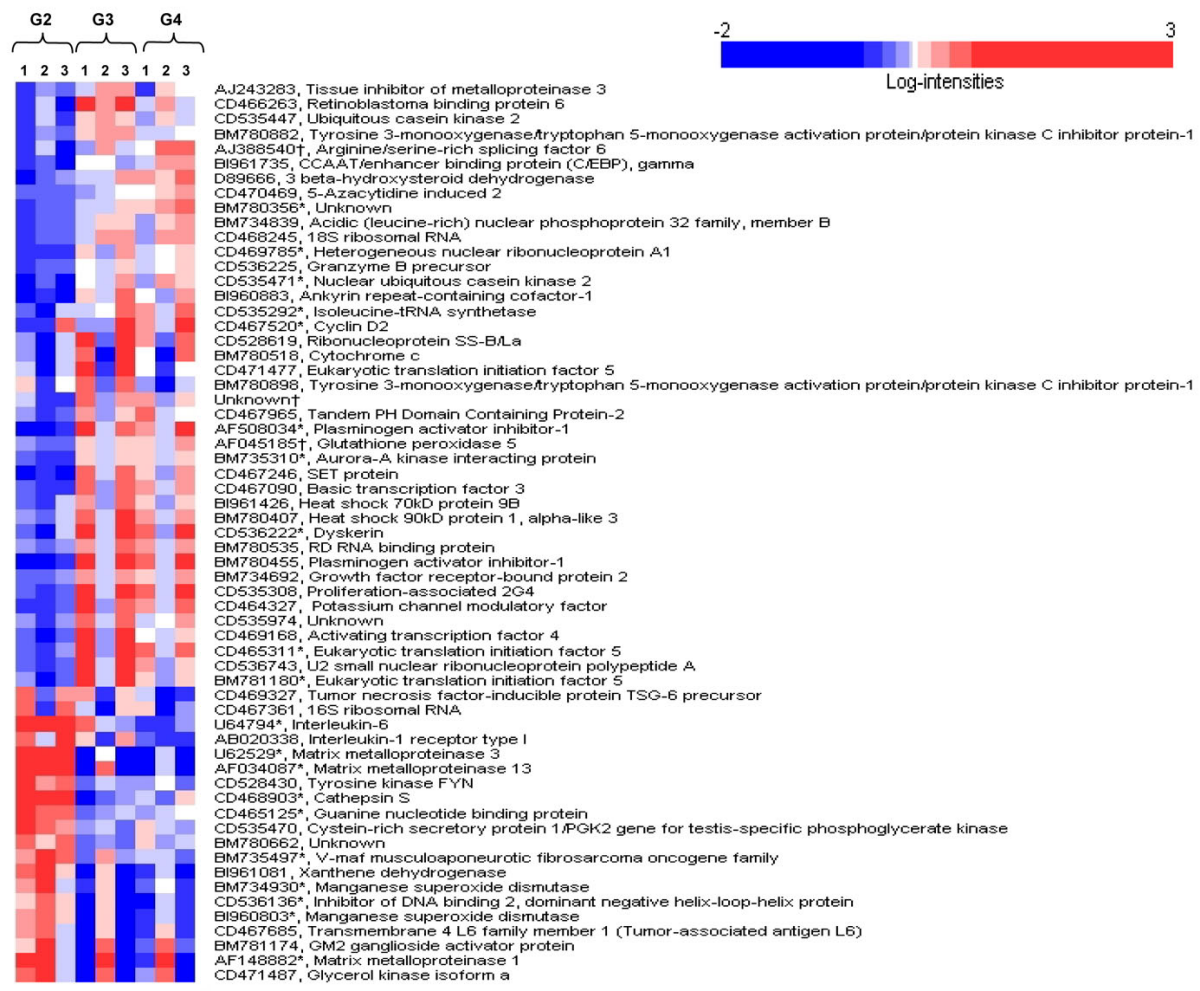
Sixty-one probe sets differentially expressed (*P *< 0.005) among the lipopolysaccharide-challenged groups. Three individual donors are represented for each group. Group 2 (G2), LPS control; group 3 (G3), pretreatment and sustained treatment with lower molecular weight hyaluronan (HA) product; group 4 (G4), pretreatment and sustained treatment with higher molecular weight HA product. Columns represent individual animals 1, 2, and 3. Rows represent probe sets ordered by a hierarchical cluster analysis using the average linkage and 1 – Pearson correlation as the measure of dissimilarity. Shading is indicative of relative expression: white, median expression; deepening shades of red, increasing expression of the probe set above the median value; deepening shades of blue, decreasing expression of the probe set below the median value. *Gene expression differentially expressed (adjusted *P *< 0.005) between at least one of the pairs of treatments. ^†^Probe set found in canines, which was included on the microarray to validate data.

A comparison of all probe sets on the microarray between groups 1 and 2 showed that 20 ng/ml LPS significantly alerted the expression of 81 probe sets (*P *< 0.005; Table 4). Sixty-one probe sets were differentially expressed among the LPS-challenged groups 2, 3, and 4 (*P *< 0.005; Figure 2). Subsequent pair-wise comparisons of these 61 probe sets were performed between groups 2 and 3 and between groups 2 and 4; a total of 19 genes represented by 21 probe sets (11 genes and 17 genes, respectively) were differentially expressed (adjusted *P *< 0.005; Table 5). No significant differences in gene expression were found between groups 3 and 4 for these 61 probe sets.

## Discussion

Our study focused on elucidating the *in vitro *effects of HA of differing molecular weights on fibroblast-like synovial cells in the face of a LPS challenge. Notably, the higher molecular weight HA product significantly reduced the morphologic change of synovial cells *in vitro *following a 2-hour challenge with 20 ng/ml LPS when compared with the lower molecular weight HA product (Table 1 and Figure 1). Higher molecular weight HA may preserve normal/healthy cell morphology due to a number of factors. Our results suggested that one probable mechanism to explain this finding is related to the ability of higher molecular weight HA to maintain hysteresis, compliance, and fluid exchange by reducing or dissipating stress associated with mechanical forces [42]. This facility may have enabled the fibroblast-like synovial cells in group 4 to resist contraction from the cell culture flasks when reacting to the cellular effects induced by LPS. It is also possible that pretreatment with the higher molecular weight HA product stimulated receptor-mediated intracellular signaling events that were not potentiated to the same degree by the lower molecular weight HA product in the presence of LPS [18,43,44]. It is worthwhile to note that a larger number of genes were statistically significantly altered by the higher molecular weight HA product (17 genes) than the lower molecular weight product (11 genes), and the degree of significance of given gene expression compared with LPS control was greater (that is, lower *P *values) with the higher molecular weight product. This is a less probable explanation, however, as no statistical difference in gene signaling was found between groups exposed to lower or higher molecular weight HA.

Other reported potential mechanisms were less supported by our data. For example, higher molecular weight HA can be more effective than lower molecular weight HA products at binding inflammatory mediators or corresponding receptors, including LPS itself and soluble agents released from challenged cells, thereby inhibiting their activity [17,39]. In particular, HA can protect surface-active phospholipids, the major boundary lubricant in joints, from lysis by phospholipase A_2 _[45]. Furthermore, it could be suggested that the higher molecular weight HA product maintained a greater degree of protection from LPS by creating an inert physical barrier not adequately provided by lower molecular weight HA. Appreciably, the comparable cell counts, PGE_2 _concentrations, and gene expression alterations of the LPS-challenged groups 3 and 4 dispute both of these theories. These results indicated that higher molecular weight HA was involved in a dynamic interaction that neither completely prevented LPS from accessing the fibroblast-like synovial cells nor bound all available LPS. Additional experimentation is warranted to fully define the mechanism behind the apparent protective effect of higher molecular weight HA relative to lower molecular weight HA upon challenge with LPS.

The gene expression profile generated by LPS challenge in this study (Table 3) was consistent with published data [35]. Addition of LPS at a concentration of 20 ng/ml induced differential expression of several genes, particularly TNFα, chondroitin sulfate proteoglycan, prostaglandin G/H synthase-2, MMP3, HA synthase 2, and IL-6. It was anticipated that there would be a reduction in the number of genes significantly altered by this concentration of LPS relative to the gene profile previously reported for 100 ng/ml by Gu and Bertone [35].

The similarity in expression profiles between the two HA-treated groups 3 and 4 suggested that differing molecular weights, within a certain range and concentration, may not be integral for initiation of intracellular signaling. The pharmacologic benefits of pretreatment and sustained treatment with exogenous HA were supported by the difference in gene expression profiles between the LPS-challenged group 2 and the HA treatment groups 3 and 4. The majority of genes that were differentially expressed between the LPS-challenged control group and the two HA treatment groups are well-recognized gene products involved with inflammatory conditions of the joint, particularly rheumatoid arthritis (Table 4) [40,41,46-53]. Additionally, we found a decrease in production of the inflammatory mediator PGE_2 _in groups 3 and 4, providing further evidence of an anti-inflammatory effect of HA. Interestingly, only two genes (IL-6 and MMP3) that were significantly increased in gene expression by LPS relative to the unchallenged control were significantly decreased in expression by the addition of HA, regardless of molecular weight (Tables 3 and 4). Other inflammatory mediators that were increased in gene expression by LPS, including TNFα, were not altered in either of the two HA treatment groups. In concert, these data suggested that pretreatment with a HA product resulted in a completely different and potentially beneficial gene expression profile when compared with the control groups. It is striking that treatment with HA shifted the gene expression profiles in an anti-inflammatory and anticatabolic direction relative to the LPS-challenged control group. Our data suggest that pretreatment and sustained exposure for 48 hours to HA may repress the molecular signaling of LPS by initiating independent intracellular events.

The limitations of this *in vitro *study with relevance to clinical application of intra-articular HA therapy are recognized. The fibroblast-like synovial cells used in this study are the largest proportion of cells found in the synovium, but are not the only component. The presence of other tissues in the joint will influence the effect of HA on overall joint homeostasis *in vivo*. In addition, the fibroblast-like synovial cells in this study were raised in either an environment devoid of HA (control groups 1 and 2) or in a stable environment containing HA (groups 3 and 4). This permitted a controlled evaluation of the influence of HA but did not mimic the *in vivo *environment of a joint, where synovial fluid is neither free of HA nor does supplemented HA permanently remain in the articular space. Future *in vivo *studies would provide important information on HA as an intra-articular therapy.

Although the mechanism of action of LPS in antibody-induced arthritis remains uncertain, its role in joint inflammation and arthritis pathogenesis is well recognized [29]. As such, our study using LPS provided further *in vitro *evidence that pre-emptive and early viscosupplementation with HA is a viable and potentially valuable treatment option for inflammatory synovitis and rheumatoid arthritis [54-56].

## Conclusion

The anti-inflammatory and anticatabolic gene expression profiles of synovial cells treated with HA and subsequently challenged with LPS supports the pharmacologic benefits of treatment with HA regardless of molecular weight. The higher molecular weight HA product provided a cellular protective effect not seen with the lower molecular weight HA product.

## Abbreviations

DMEM-S = Dulbecco's modified Eagle medium supplemented with 10% fetal bovine serum, 29.2 mg/ml L-glutamine, 50 U penicillin/ml, and 50 U streptomycin/ml; ELISA = enzyme-linked immunosorbent assay; HA = hyaluronan; IL = interleukin; LPS = lipopolysaccharide; MMP3 = matrix metalloproteinase 3; PCR = polymerase chain reaction; PGE_2 _= prostaglandin E_2_; RT = reverse transcriptase; TNFα = tumor necrosis factor alpha.

## Competing interests

The authors declare that this study was funded, in part, by Pfizer Animal Health, Inc.

## Authors' contributions

KSS performed the cell culture work, the ELISAs, and some RNA extractions, and drafted the manuscript. ALJ performed the majority of the RNA extractions. ASR contributed to the statistics involved in the gene expression analysis. ALB conceived of and coordinated the study, edited the manuscript, and obtained funding for the project. All authors read and approved the final manuscript.

## Figures and Tables

**Table 1:** Microscopic variables for fibroblast-like synovial cells

	Day 1 (pre-lipopolysaccharide challenge)				Day 1 (post-lipopolysaccharide challenge)				Day 2			
	
	Group 1	Group 2	Group 3	Group 4	Group 1	Group 2	Group 3	Group 4	Group 1	Group 2	Group 3	Group 4
Cellular morphology (median and range)	0 (0-0)	0 (0-0)	0 (0-0)	0 (0-0)	0 (0-0)	3^a ^(0–4)	4^a ^(3–4)	1 (0–4)	0 (0-0)	3^b ^(0–4)	4^b ^(3–4)	0 (0–4)
Cell count (10^4^) (mean ± SEM)	20	20	20	20	-	-	-	-	118^c ^± 33	46 ± 3	32 ± 1	49 ± 7
Cell viability (%) (mean ± SEM)	-	-	-	-	-	-	-	-	97 ± 0.3	96 ± 1.2	94 ± 2.6	96 ± 0.1

Triplicate samples were performed for each of the three individual donors in the four groups. Group 1, unchallenged control; group 2, lipopolysaccharide control; group 3, pretreatment and sustained treatment with lower molecular weight hyaluronan product; group 4, pretreatment and sustained treatment with higher molecular weight hyaluronan product. 0, >95% attached; 1, 5–25% detached; 2, 26–50% detached; 3, 51–75% detached; 4, >76% detached. SEM, standard error of the mean; -, values not determined. ^a,b,c^Significant difference exists (*P *< 0.05).

**Table 2:** Mean concentrations of prostaglandin E_2_, IL-6, and matrix metalloproteinase 3 in culture media of fibroblast-like synovial cells determined by ELISAs

	Group 1	Group 2	Group 3	Group 4
Prostaglandin E_2 _(pg/ml)	15.43 ± 15.43	21,025^a ^± 6,828	2,998^b ^± 887	2925^b ^± 1,669
IL-6 (pg/ml)	1.77 ± 1.74	41.33^c ^± 16.18	9.49 ± 3.53	6.79 ± 3.11
Matrix metalloproteinase 3 (pg/ml)	2.60 ± 1.36	6.31^d ^± 0.26	2.96 ± 1.08	1.80 ± 0.90

Data presented as the mean ± standard error of the mean. Triplicate samples were performed for each of the three individual donors in the four groups. Group 1, unchallenged control; group 2, lipopolysaccharide control; group 3, pretreatment and sustained treatment with lower molecular weight hyaluronan product; group 4, pretreatment and sustained treatment with higher molecular weight hyaluronan product. ^a,b^Significant difference exists (*P *< 0.05). ^c,d^Significant difference exists (*P *< 0.01).

**Table 3:** Comparison of the fold changes between species-specific microarray analysis and real-time RT-PCR performed on fibroblast-like synovial cells exposed to lipopolysaccharide

	Fold change after 100 ng/ml lipopolysaccharide challenge		Fold change after 1,000 ng/ml lipopolysaccharide challenge		Real-time RT-PCR primer
		
	Microarray analysis	Real-time RT-PCR	Microarray analysis	Real-time RT-PCR	
IL-1α	16	155	34	290	Forward, TTGTGCCAACCAATGAGATCA
					Reverse, TTCATGCTTTGCCTTCTTCTTG
TNFα	5	6	6	10	Forward, GACTTGAAGTTTTCTAAGCGATGCT
					Reverse, GGATCCACTGCCACGTACTTG
Prostaglandin peroxide synthase	3	2	3	4	Forward, GGCCAGTTTTCCTCACCAAA
					Reverse, AAATAAAGCTCTCTGCTTTTCATGAA

The fold changes are normalized to unchallenged fibroblast-like synovial cells.

**Table 4:** Select/relevant genes with significant differential gene expression between the lipopolysaccharide-challenged control group and the unchallenged control group

Genbank accession number	Equine gene	Fold change (group 2/group 1)	*P *value
AY114351	Granulocyte chemotactic protein 2	10.0	<0.0001
CD528275	Interferon-induced protein	0.03	<0.0001
CD468799	GRO3 oncogene	10.0	<0.0001
BI960809	TNFα	2.5	0.0001
AJ251189	Chemoattractant protein-1	3.33	0.0016
CD536631	GRO2 oncogene	10.0	0.0002
BM734848	Chondroitin sulfate proteoglycan 2	2.0	0.0005
AB035518	Adrenomullin	0.42	0.0006
AF230359	Urokinase plasminogen activator receptor	1.67	0.0009
AF053497	Melanoma growth stimulatory activity homolog	5.0	0.0014
AF027335	Prostaglandin G/H synthase-2	3.33	0.0021
U62529	Matrix metalloproteinase 3	2.0	0.0026
CD467591	Heat shock protein 70	2.5	0.0027
AY056582	Hyaluronic acid synthase 2	5.0	0.0028
U64794	IL-6	2.0	0.0037

Three individual donors are represented for each group. Group 1, unchallenged control; group 2, lipopolysaccharide-challenged control.

**Table 5:** Genes significantly upregulated or downregulated in lipopolysaccharide-challenged groups 2, 3, and 4

Full or provisional gene annotation (accession number)	Function or activity in joint inflammation^a^	Fold change		Pair-wise comparisons (adjusted *P *values)	
		
		Group 3/group 2	Group 4/group 2	Group 3 vs group 2	Group 4 vs group 2
IL-6 (U64794)	Proinflammatory mediator [46]	0.45	0.34	**0.0015**	**0.0015**
Matrix metalloproteinase 13 (AF034087)	Connective tissue structure and remodeling [40]	0.10	0.11	**0.0039**	**0.0005**
Cathepsin S (CD468903)	Proteolysis and matrix degradation [47]	0.47	0.57	**0.0022**	**0.0005**
Manganese superoxide dismutase (BM734930)	Antioxidant [48]	0.62	0.71	**0.0040**	**0.0040**
Manganese superoxide dismutase (BI960803)		0.50	0.64	0.0117	**0.0024**
Matrix metalloproteinase 1 (AF148882)	Connective tissue structure and remodeling [40]	0.32	0.35	**0.0050**	**0.0003**
Matrix metalloproteinase 3 (U62529)	Connective tissue structure and remodeling [40]	0.24	0.19	**0.0035**	0.0051
Guanine nucleotide binding protein alpha inhibiting 1 (CD465125)	G-protein signaling, adenylate cyclase inhibitor [49]	0.62	0.66	0.0079	**0.0046**
V-maf oncogene (BM735497)	Unknown	0.60	0.59	**0.0035**	0.0095
Inhibitor of DNA binding 2 dominant negative helix–loop–helix protein (CD536136)	Positive regulation of cell proliferation [50]	0.60	0.66	0.0172	**0.0038**
Plasminogen activator inhibitor 1 (BM780455)	Inhibitor of proteolytic activity in rheumatoid arthritis [51,52]	2.93	2.59	**0.0002**	**<0.0001**
Plasminogen activator inhibitor 1 (AF508034)		2.11	2.15	**0.0025**	**<0.0001**
hnRNP core protein A1 (CD469785)	Target of antinuclear autoimmunity in rheumatoid arthritis [41]	1.41	1.40	0.0114	**0.0028**
Aurora-A kinase interacting protein 1 (BM735310)	Positive regulator of proteolysis	1.31	1.35	0.0215	**0.0044**
Dyskerin (CD536222)	RNA binding, processing, and modification	2.30	1.74	**0.0046**	**0.0028**
Cyclin D_2 _(CD467520)	Induced by type I interferons after lipopolysaccharide exposure; cell cycle regulation	1.24	1.50	0.0500	**0.0020**
Isoleucine tRNA synthetase (CD535292)	Isoleucyl-rRNA aminocylation	1.36	1.50	0.0254	**0.0015**
Nuclear ubiquitous casein kinase 2 (CD535471)	Kinase in NF-κB cascade [53]	1.62	1.55	0.0294	**0.0048**
Eukaryotic translation initiation factor 5 (BM781180)	Translation initiation factor	1.95	1.33	**0.0048**	0.0050
Eukaryotic translation initiation factor 5 (CD465311)		2.26	1.55	0.0146	**0.0013**
Unknown (BM780356)	Unknown	1.28	1.55	0.0316	**0.0011**

Three individual donors are represented for each group. ^a^Based on the Gene Ontology Database description and the literature (see references). Statistically significant *P *values are in bold. Group 2, lipopolysaccharide control; group 3, pretreatment and sustained treatment with lower molecular weight hyaluronan product; group 4, pretreatment and sustained treatment with higher molecular weight hyaluronan product.
